# Towards a methodological uniformization of environmental risk studies in Parkinson’s disease

**DOI:** 10.1038/s41531-024-00709-y

**Published:** 2024-04-17

**Authors:** Bruno Lopes Santos-Lobato

**Affiliations:** Laboratório de Neuropatologia Experimental, Universidade Federal do Pará, Belém, Brazil

**Keywords:** Parkinson's disease, Risk factors

In a recent Brief Communication, Payami et al. ^1^ explored the impact of specific risk factors on the onset of Parkinson’s disease (PD), including the heavy use of pesticides in an underrepresented region of the United States, the Deep South. Further, they represented these associations through the population attributable fraction (PAF), which shows the percentage of preventable new PD cases if risk factors were eliminated. They showed the heavy use of pesticides increased the risk of developing PD, with PAF of 17% in males and 23% in females. The results mean that approximately one in five cases of PD would be prevented if people had no heavy pesticide exposure.

Other questionnaire-based studies also explored this association. Analyzing people living in a developing country (Brazil), we described that high exposure to household pesticides (defined as >30 days per year) was associated with higher odds of developing PD (adjusted odds ratio = 2.27, 95% confidence interval 1.46-3.52) in a total sample of 936 participants^2^. Considering that synthetic pyrethroid insecticides are the main household pesticides globally used for pest control of mosquito-borne diseases, the rational use of these chemical products might reduce the number of new PD cases.

To estimate the percentage of PD cases potentially prevented if the high exposure to household pesticides were eliminated, we used the same methodology described by Payami et al. (Miettinen formula)^3^ to calculate the PAF in our data. With a prevalence of 18% in persons with PD and high household pesticide exposure, the overall PAF was 10% (5–12%), with minimal variation according to sex (Table 1). Additionally, a previous study from California showed that the frequent use of household pesticides increased the risk of developing PD^4^. For these results, the PAF of frequent use of household pesticides was 9% (3–16%) (Table 1). Together, these studies indicate that the elimination of excessive use of pesticides may reduce 10–20% of new PD cases.

**Table 1:** Population attributable fraction for excessive household pesticide exposure in different studies

Author, Year	Total			Multivariable analysis					Population attributable fraction		
	Group	PD	Controls	N with data		PD vs. Controls			Determinants of PAF		PAF (95% CI)
				PD	Controls	Adj OR	95% CI	p-value	Adj OR	Prev. in PD	
**Payami et al**. ^1^	**Males**	512	125	297	88	2.52	1.37-?	< 0.001	2.52	28%	17% (7-27%)
	**Females**	296	290	193	206	2.85	1.87-?	< 0.001	2.85	35%	23% (12-34%)
**Moura et al**. ^2^	**All participants**	562	374	539	356	2.27	1.46-3.52	< 0.001	2.27	18%	10% (5-12%)
	**Males**	293	165	282	156	2.19	1.18-4.04	0.012	2.19	20%	10% (3-15%)
	**Females**	269	209	257	200	2.43	1.28-4.6	0.006	2.43	16%	9% (3-12%)
**Narayan et al**. ^4^	**All participants**	357	807	357	807	1.37	1.13-1.92	Not described	1.37	34%	9% (3-16%)

Household pesticide exposure was tested for association with PD through a multivariate logistic regression analysis (described in the original studies), followed by PAF calculation for exposure. *Adj OR* adjusted odds ratio, *CI* confidence interval, *PAF* population attributable fraction, *PD* Parkinson’s disease, *Prev*. prevalence.

Despite the similarity of these results, the heterogeneity of methodologies adopted by these questionnaire-based studies is an issue and may hamper the interpretation of data. Aiming to improve the accuracy of these assessments, we propose researchers in this field to uniformize methodology on three topics: (I) method of measurement of pesticide exposure, (II) definition of high pesticide exposure, and (III) statistical analysis.

Regarding the methods of measurement of pesticide exposure, each of these three studies applied distinct questionnaires, with merits and flaws. For example, the analyses of Moura et al. used data from a short questionnaire proposed by the Latin American Research consortium on the GEnetics of Parkinson’s Disease (LARGE-PD) which did not distinguish among different pesticide chemical classes and age categories but included a high-tier frequency category of high threshold (use >30 days per year)^2^. Payami et al. used a questionnaire with no discrimination of household or occupational pesticide use or distinct exposure to herbicides, insecticides, and fungicides, and defined categories of frequency of use^1^. Narayan et al. used a more complex instrument to register data regarding different environments (occupational vs. household), pesticide chemical classes, and age categories but with a high-tier frequency category of low threshold (use once a month or more)^4^. The existence of a high-tier frequency category with a high threshold (once a week, more than once a week) in these questionnaires is particularly important for measuring exposure in developing countries, considering their vulnerability to incorrect use of these products and informal market^5^.

The Parkinson’s Disease Risk Factor Questionnaire (PDRFQ)^6^ is a tool for collecting lifelong health information of people with PD and has been adopted by large-size PD studies, such as the Parkinson’s Progression Markers Initiative (PPMI) and the Fox Insight. For household pesticide use, the questions are categorized by type (insecticides, fungicides, herbicides) and age, including information about safety measures and acute intoxications. As a flaw, the high-tier frequency category has a low threshold (use six times a year or more). Thus, we suggest the use of the PDRFQ for pesticide risk studies, but with a high threshold high-tier frequency category, as in the LARGE-PD questionnaire (1–5 days/year, 6–10 days/year, 11–30 days/year, >30 days/year).

The definition of high pesticide exposure was variable among these studies. Moura et al. arbitrarily considered each participant who used pesticides >30 days per year as high exposure^2^. Payami et al. asked people if they had been “ever exposed to heavy uses of pesticides or herbicides”, without a clear definition of “heavy use”^1^. Narayan et al. estimated the lifetime exposure with calculations with frequency category and years of use, and the high pesticide exposure was defined as a frequency equal to or higher than the median of estimated exposure^4^. For uniformization, we also suggest an estimate-based definition of high pesticide exposure as proposed by Narayan et al. and a recent study exploring the impact of household pesticides on the progression of PD^7^.

All studies performed multivariate logistic regressions for statistical analysis to calculate the odds ratio and 95% confidence intervals. The selection of covariates used for adjusting in multivariate models for the association studies of pesticide exposure and the onset of PD is essential for the goodness-of-fit. For example, lifetime smoking, a protective factor for PD onset, is a common covariate included in the multivariate regression model of these studies^2,4^. Thus, we propose the inclusion of relevant covariates for the onset of PD (sex, age at evaluation/onset, origin, smoking, education, family history, polygenic risk score) for these models.

In conclusion, more studies exploring the association between excessive use of pesticides and the onset of PD in different populations and socioeconomic backgrounds are needed, including questionnaire-based evaluations and other strategies of analyses (land use of pesticides, measurement of pesticide metabolites in human samples). However, the methodology of collecting data about pesticide exposure must be less heterogeneous among studies. A larger number of studies may help better define harmful levels of pesticide exposure, aiming at a rational use of these substances.

## Data Availability

Data sharing not applicable to this article as no datasets were generated or analysed during the current study.
